# Adverse socioeconomic factors are associated with a widening gap in one-year health-related quality of life after acute myocardial infarction

**DOI:** 10.1038/s41598-025-04604-1

**Published:** 2025-06-05

**Authors:** David Füller, Henrike Andresen-Bundus, Nikolaos Pagonas, Philipp Jaehn, Christian Ukena, Kathrin Gödde, Christine Holmberg, Oliver Ritter, Benjamin Sasko

**Affiliations:** 1 Brandenburg Medical School (Theodor Fontane), Neuruppin, Brandenburg an der Havel, Germany; 2 Division of Cardiology, Department of Internal Medicine, University Hospital Brandenburg an der Havel, Brandenburg Medical School (Theodor Fontane), Brandenburg an der Havel, Germany; 3 Division of Cardiology, Medical Department A, University Hospital Ruppin-Brandenburg, Brandenburg Medical School (Theodor Fontane), Neuruppin, Germany; 4 Institute of Social Medicine and Epidemiology, Brandenburg Medical School (Theodor Fontane), Brandenburg an der Havel, Germany; 5 Ruhr-University of Bochum, Medical Department II, Marien Hospital Herne, Hölkeskampring 40, 44625 Herne, Germany

**Keywords:** Acute coronary syndrome, Health-related quality of life, EQ-5D-5L, Socioeconomic factors, Clinical presentation, Disease prevention, Health policy, Public health, Quality of life

## Abstract

Acute myocardial infarction (AMI) has a significant impact on the health-related quality of life (HRQoL) and is influenced by unfavorable socioeconomic factors. We aimed to evaluate the association between adverse socioeconomic factors including low educational level, low occupational qualification and financial hardship with presenting symptoms and HRQoL in patients hospitalized for an AMI. We hypothesized a detectable effect on a spatial level and therefore assessed the HRQoL in 298 patients with AMI using the EQ-5D-5L generic measure for health status. Sociodemographic characteristics, clinical data, medical history, and prevalence of cardiovascular risk factors were obtained. Self-reported HRQoL was determined upon hospital admission and after 12 months. Patients with lower educational attainment were more likely to report dyspnea at hospital admission, had a worse renal function and more frequently hypertension. One year post-AMI the health state of the cohort worsened in terms of the mobility, activity, pain, and anxiety/depression domains but not the self-care domain. Patients with a lower education level or a poor financial situation reported a worse HRQoL and health state at baseline and follow-up (change in EQ VAS baseline—follow-up − 7.7 and − 11.0, respectively, lowest category). In contrast, the health state of patients with a higher education level or better financial situation improved (change in EQ VAS baseline—follow-up + 4.0 and + 2.6, highest category). Our study demonstrates a substantially widening gap in the health state and HRQoL between patients with lower and higher educational attainment within the first year after AMI, already measurable on a spatial level.

## Introduction

Acute myocardial infarction (AMI) is among the leading causes of death worldwide and the fourth leading cause of death in Germany with significant differences in its morbidity and mortality on regional and local levels^1–4^. In 2019, the risk of dying from an AMI was more than twice as high in the federal state of Brandenburg than in the federal state of Schleswig–Holstein. Although both states have a comparable rural character, age-standardized mortality rates varied between 106 and 178 deaths per 100,000 within the same year^4^.

This effect might be linked to socioeconomic factors: using the German Index of Socioeconomic Deprivation (GISD) to assess spatial socioeconomic deprivation (SSD), we have previously investigated that the hospitalization rates for AMI are 29% lower in areas of the immediate vicinity of the city of Brandenburg an der Havel if compared to the city of Brandenburg an der Havel itself, which is characterized by a higher SSD than surrounding areas^5^. Spatial socioeconomic deprivation comprises individual opportunities for social participation that are negatively influenced by socio-spatial circumstances.

Comparative studies which investigate the underlying mediating factors to the association between area of residence and AMI mortality risk in Germany are lacking. Moreover, the significance of adverse individual-level socioeconomic factors including low educational level, low occupational qualification, and financial hardship for cardiovascular-health related outcomes including clinical presentation and health-related quality of life (HRQoL) in a population with high spatial socioeconomic deprivation has not been previously investigated in Germany.

Individual-level socioeconomic factors can influence HRQoL in patients with coronary heart disease and AMI as these factors can affect access to healthcare, adherence to treatment, and psychosocial well-being. SSD can exacerbate health disparities and contribute to poorer HRQoL outcomes in these patients through limited access to healthcare and specialists leading to delayed treatment, and as it can be associated with lower health literacy, more social isolation and fewer support networks.

A study from Jordan found an association between lower income and HRQoL in patients affected from AMI^6^. Other smaller studies have had similar findings, however, all with smaller sample sizes and outside Europe, thus, with different cultural and religious backgrounds. The relationship between changes in HRQoL, occupational qualification or income/financial situation has not yet been investigated in patients with AMI. The association between level of education and HRQoL has been previously investigated, but with different results^7–9^.

We hypothesized that there are differences in health status by socioeconomic factors both before admission for an AMI and one year post-AMI. Therefore, in this study, we aimed to (a) investigate the prevalence of adverse socioeconomic factors including low educational level, low occupational qualification, and financial hardship in a cohort of patients undergoing emergent coronary angiography for an AMI at the university hospital Brandenburg. We further aimed to (b) determine the association and possible intra-individual changes of the HRQoL in relation to these socioeconomic factors. Finally, we aimed to (c) investigate potential differences in initial clinical variables and presentation in the hospital by educational attainment. Education is a central indicator of socioeconomic status which is associated with health literacy and that was previously hypothesized to impact medical care in AMI, thus, may affect time until presentation and outcomes in this population^10^. Previous studies have demonstrated differences in clinical outcomes in AMI based on level of education including heart failure, pulmonary edema, shock, and renal failure^11,12^.

## Methods

The present study includes patients from the Myocardial Infarction Registry Brandenburg (“Herzinfarktregister Brandenburg”)^13^. Between 4/2019 and 12/2020, 572 individuals undergoing emergent coronary angiography due to an AMI were consecutively recruited into this single-center prospective study in rural-suburban eastern Germany. Our hospital is located in a region with high SSD which has one of the highest AMI mortality rates in Germany. Patients who survived the initial 24 h were asked to participate in this study. Furthermore, as part of the inclusion criteria, potential participants were asked for their willingness to provide detailed sociodemographic information. This resulted in a total of 298 patients for study inclusion. The excluded patients (N = 274) could not be included for various reasons, which included the unwillingness to participate, altered mental state, e.g. presence of dementia or delirium, admission to ICU with invasive or non-invasive ventilation or organ replacement procedures, urgent transfer for bypass surgery, need for isolation due to an infectious disease such as COVID-19 or influenza, admission at the weekend and prompt discharge before a study nurse could interview the patient, and death of the patient or poor clinical condition.

The first patient interview was conducted during the day on weekdays in the cardiology ward. The follow-up interview was carried out by telephone one year later by an interviewer, provided the patients had given written consent for the follow-up.

Prevalence of previous diagnosis, existent cardiovascular risk factors and medication intake was recorded. Weight and height were measured, followed by a calculation of the body mass index (BMI, kg/m^2^). Estimated glomerular filtration rate (eGFR) (mL/min/1.73 m^2^) was computed using the Chronic Kidney Disease Epidemiology Collaboration equation during data analysis^14^.

Sociodemographic characteristics were obtained by interview of a total of 298 patients using a self-developed structured, standardized questionnaire and an additional chart review. The study protocol of the Myocardial Infarction Registry Brandenburg was published before^13^. For a detailed assessment of social determinants, a questionnaire was developed in cooperation with the Robert Koch-Institute, Berlin, Germany, and the Brandenburg State Office for Occupational Safety, Consumer Protection and Health (“Landesamt für Arbeitsschutz, Verbraucherschutz und Gesundheit”) to assess level of education, occupational position, and income. All instruments used in the Myocardial Infarction Registry Brandenburg align with previous studies and projects including the MONICA/KORA project^15^, the RHESA study^16^, or the questionnaire used in the German health update study (GEDA) 2014/2015 European Health Interview Survey (EHIS)^17^.

Level of education and occupational qualification were assessed based on the highest qualification achieved, respectively, and obtained by self-report. For the level of education: 1. No more than seven years of school attendance, elementary or secondary school, no school-leaving certificate. 2. Higher secondary school. 3. A-levels. For the occupational qualification: 1. No occupational qualification. 2. Vocational education and training. 3. College education.

Due to a very low response rate for actual annual household income among the first patients included in the registry, we designed an alternative question about financial situation, tailored to the study population of both younger and older adults, to better assess their economic background. This alternative question regarding participants’ financial situation was used in the present study. The financial situation was recorded by self-assignment to one of three categories: 1. Very good or good. This means: a reliable and regular above-average income for at least two years at the time of hospitalization (e.g. through a permanent job, which is usually not temporary, or other income such as rental income, successful self-employment, etc.). In addition, there was no fear of financial difficulties. For pensioners: e.g. own home and good pension. 2. Satisfactory or sufficient. This means: a reliable, regular and satisfactory or sufficient income from work or other sources at the time of hospitalization. No constant or recurring fear of financial difficulties. If necessary, financial help could be provided by relatives. For pensioners: sufficient pension. 3. Insufficient or financial worries. This means: an unreliable or irregular income at the time of hospitalization, e.g. due to job insecurity or financial worries or anxiety, or an income that is insufficient or too low for your needs.

The EQ-5D-5L is a generic measure of health status, introduced and provided by the EuroQol Group Association (EuroQol Research Foundation, Rotterdam, The Netherlands). It was designed for use in observational studies, population health surveys, outcome measurements, clinical trials, and other types of studies, particularly in measuring social inequalities in self-reported health. EQ-5D-5L index values can be used to estimate quality-adjusted life year gains in economic evaluations of healthcare interventions. The EQ-5D-5L consists of the EQ-5D five dimensional descriptive system and the EQ visual analogue scale (EQ VAS). The EQ-5D descriptive system comprises information on the following five dimensions: mobility, self-care, usual activities, pain/discomfort, and anxiety/depression. Each dimension has five levels ranging from no problems to extreme problems/unable to (one to five). The five dimensions are aggregated into a five-digit number. The EQ VAS is a quantitative measure of the patients’ self-perceived health and is ranked from one to 100, with 100 indicating the best health the participant could imagine. From the EQ-5D descriptive system, an EQ-5D-5L index value is calculated based on a value set which provides values (weights) for each health state description. A specific value set for Germany has been developed to allow for population preference-based HRQoL measurements^18^. The valuation study was designed to create an index value between zero and one (zero indicating a health status equal to death, and one indicating “full health”) but can include index values below zero (indicating a health status worse than being dead). The available modes of administration include a self-complete on paper questionnaire and a telephone administered version. The EQ-5D-5L was administered in compliance with the user guide and instructions provided on the EuroQol website (https://euroqol.org/).

One-year follow-up of the patients was conducted via telephone interview and was blinded for the socioeconomic variables provided at baseline for the interviewer.

The local ethic committee at the Brandenburg Medical School (Theodor Fontane) approved the study (Ref. E-01-20200923). The study was performed in accordance with the Declaration of Helsinki and its later amendments. Informed consent was obtained from all patients.

### Statistical analysis

All socioeconomic variables were treated as categorical, with the lowest levels of education, occupational status, and the poorest financial situation serving as reference categories. Shapiro–Wilk test was used to test the normal distribution assumption of metric variables. If the normal distribution assumption was not rejected, the F test was used to compare values across different categories of educational attainment, occupational qualification, and the financial situation. If the normal distribution assumption was rejected, Kruskal–Wallis test was used. For categorical variables, Chi-square and Fisher’s exact test were used where appropriate to compare the frequency distribution of a variable from independent groups. Bowker’s test for symmetry was used to compare the frequency distribution of a categorical variable from dependent groups.

The EQ-5D-5L was evaluated in compliance with the user guide and instructions provided on the EuroQol website (https://euroqol.org/). Analyses were conducted using SAS 9.3 and Stata/IC 16.0.

## Results

Baseline characteristics of the cohort are shown in Table 1. The mean age of the cohort was 66.6 ± 13.0 years and 28.2% of the participants were female. 36.0% of participants had a low educational level. Only 18.8% of participants had graduated in high school (high education level). Almost 75% of participants had vocational education and training (intermediate occupational qualification), whereas a smaller proportion had either no occupational qualification (11.3%) or a university education or degree (17.1%). 11.8% of participants self-reported that their perceived financial situation was insufficient, and 33.8% stated that it was good or very good.

**Table 1:** Baseline characteristics of the study cohort (N = 298).

Variable			Missing values
Age (years)		66.6 ± 13.0	
Sex	Male	71.8%	
	Female	28.2%	
Leading diagnosis upon discharge	STEMI	34.2%	
	NSTEMI	64.4%	
	Non-Type 1 AMI	1.4%	
Hypertension		73.5%	
Diabetes		33.9%	
Smoking	Never smoker	42.7%	3
	Former smoker	21.7%	
	Current smoker	35.6%	
History of cardiovascular disease^a^		44.3%	
Way of referral to hospital with cardiac catheterization laboratory	Self-presentation	12.9%	3
	General Practitioner	20.0%	
	Emergency Medical Services	59.7%	
	Referred from another hospital	7.5%	
BMI (kg/m^2^)		28.4 ± 4.9	
eGFR (mL/min/1.73 m^2^)		72.3 ± 22.5	
HDL (mmol/L)		1.16 ± 0.31	17
LDL (mmol/L)		3.10 ± 1.15	17
Total cholesterol (mmol/L)		4.71 ± 1.29	42
Triglycerides (mmol/L)		1.95 ± 1.20	18
Lipoprotein a (mmol/L)		34.5 ± 52.4	58
Max CK (U/L)		839.7 ± 1185.3	
NT-proBNP (pg/mL)		2635.6 ± 4670.0	55
CRP (mg/L)		12.7 ± 28.5	1
Glucose (mmol/L)		8.9 ± 4.0	2
Urea (mmol/L)		6.7 ± 3.8	64
HbA1c (%)		6.2 ± 1.4	47
Educational attainment	No more than seven years of school attendance, elementary or secondary school, no school-leaving certificate	36.0%	6
	Higher secondary school	45.2%	
	A-levels	18.8%	
Occupational qualification	No occupational qualification	11.3%	5
	Vocational education and training	71.7%	
	College education	17.1%	
Financial situation	Insufficient	11.8%	103
	Satisfactory or adequate	54.4%	
	Good/very good	33.8%	
Time of follow up (days)		387 ± 47	
Loss of follow up		29.9%	
EQ-5D index value BL		0.86 ± 0.26	
EQ-5D index value FU		0.79 ± 0.27	103
Change EQ-5D index BL-FU		−0.10 ± 0.27	103
EQ VAS BL		68.5 ± 20.2	
EQ VAS FU		68.6 ± 21.8	105
Change EQ VAS BL-FU		−2.3 ± 24.3	109

^a^History of cardiovascular disease incudes prior ischemic heart disease, valvular pathology, heart failure, stroke, peripheral artery disease, and cardiac arrhythmia.

Mean ± standard deviation shown unless indicated. STEMI, ST-elevation myocardial infarction. NSTEMI, Non-ST-elevation myocardial infarction. BMI, body mass index. eGFR, estimated glomerular filtration rate. HDL, high-density lipoprotein cholesterol. LDL, low-density lipoprotein cholesterol. Max CK, maximum creatine kinase (indicating the highest level of creatine kinase measured in plasma). NT-proBNP, N-Terminal pro-Brain Natriuretic Peptide. CRP, C-reactive protein. BL, baseline. FU, Follow up.

Differences in clinical variables and presentation by educational level are displayed in Table 2. There were no differences in the time of onset of symptoms to first medical contact, time of most severe chest pain or discomfort to arrival in the emergency department, and door-to-wire-times in STEMI and NSTEMI by educational level. Patients with lower educational attainment were more likely to report difficulties breathing at admission in the hospital. Furthermore, they had worse renal function and were more likely to have a history of hypertension.

**Table 2:** Differences in clinical variables and presentation by educational level (N = 294).

Variable	Group 1 N = 106	Group 2 N = 133	Group 3 N = 55	*P*
Age (years)	74.0 ± 12.3	60.4 ± 10.7	67.1 ± 12.4	< 0.001
Time of onset of symptoms to first medical contact (hours) (median [IQR])	2.8 [23.0]	4.2 [26.5]	3.0 [27.0]	0.63
Time of most severe chest pain or discomfort to arrival in the emergency department (hours) (median [IQR])	1.5 [2.5]	2.0 [2.4]	1.4 [4.2]	0.17
Door-to-wire-time STEMI (hours) (median [IQR])	0.8 [1.1]	0.8 [1.1]	0.5 [0.7]	0.28
Door-to-wire-time NSTEMI (hours) (median [IQR])	13.4 [23.2]	11.8 [17.0]	16.7 [20.0]	0.34
Chest pain at admission (%)	86.8	93.2	94.5	0.14
Difficulty breathing at admission (%)	57.5	56.4	38.2	0.04
Syncope prior to admission (%)	5.7	3.8	5.5	0.76
Signs of heart failure at admission (%)	16.0	9.0	14.5	0.24
Cardiogenic shock at admission (%)	1.9	3.0	1.8	0.88
Resuscitation before or at admission (%)	0.9	3.0	3.6	0.48
Vessels with significant stenosis identified in PCI (%)^a^				
1	23.8	27.7	31.5	0.88
2	32.4	32.3	29.6	
3	43.8	40.0	38.9	
Vessel causing AMI after PCI (%)^a^				
TIMI 0–2	13.9	7.7	3.8	0.09
TIMI 3	86.1	92.3	96.2	
Max CK (U/L)^a^	639.9 ± 803.8	1025.2 ± 1416.7	805.4 ± 1122.3	0.11
CRP (mg/L)^a^	15.1 ± 35.8	11.1 ± 22.7	10.6 ± 25.5	0.11
eGFR (mL/min/1.73 m^2^)^a^	63.5 ± 22.9	80.4 ± 20.3	70.0 ± 19.9	< 0.001
EF (%)^a^	51.3 ± 12.6	53.8 ± 12.0	54.5 ± 10.1	0.2
BMI (kg/m^2^)^a^	28.0 ± 5.2	28.6 ± 5.0	28.7 ± 4.3	0.47
Hypertension (%)^a^	81.7	70.0	68.5	0.008
Diabetes (%)^a^	40.4	27.7	33.3	0.12
Smoking (%)^a^				
Never smoker	53.8	29.2	51.9	< 0.001
Former smoker	18.3	23.1	27.8	
Current smoker	27.9	47.7	20.4	
Hyperlipoproteinemia (%)^a^	73.1	63.8	66.7	0.32

Group 1: No more than seven years of school attendance, elementary or secondary school, no school-leaving certificate. Group 2: Higher secondary school. Group 3: A-levels.

^a^Only those patients with STEMI or NSTEMI as the leading diagnosis upon discharge are included in this analysis.

Mean ± standard deviation shown unless indicated. IQR, inter quartile range. STEMI, ST-elevation myocardial infarction. NSTEMI, Non-ST-elevation myocardial infarction. PCI, percutaneous coronary intervention. TIMI, Thrombolysis in myocardial infarction-classification. Max CK, maximum creatine kinase (indicating the highest level of creatine kinase measured in plasma). CRP, C-reactive protein. eGFR, estimated glomerular filtration rate. EF, ejection fraction. BMI, body mass index.

Baseline values of the self-reported health state of the cohort are displayed in Table 3. In 195 patients who have a complete health state available both at baseline and at follow-up, changes in the health state based on the five dimensions of the EQ-5D-5L were evaluated. One year post-AMI, the health state of the cohort worsened in terms of the mobility, activity, pain, and anxiety/depression domains but not the self-care domain (Table 4).

**Table 3:** Baseline values of the health state of the cohort (N = 298).

Variable	Value	%
Mobility	No problems	67.1
	Slight problems	18.1
	Moderate problems	9.1
	Severe problems	5.0
	Unable to walk around	0.7
Self-care	No problems	81.5
	Slight problems	9.1
	Moderate problems	5.4
	Severe problems	2.7
	Unable to wash or dress	1.3
Activity	No problems	73.8
	Slight problems	12.4
	Moderate problems	8.1
	Severe problems	4.4
	Unable to do usual activities	1.3
Pain	No pain/discomfort	59.4
	Slight pain/discomfort	20.5
	Moderate pain/discomfort	12.4
	Severe pain/discomfort	5.7
	Extreme pain/discomfort	2.0
Anxiety	Not anxious/depressed	71.8
	Slightly anxious/depressed	16.1
	Moderately anxious/depressed	6.7
	Severely anxious/depressed	3.0
	Extremely anxious/depressed	2.3

**Table 4:** Health state of the cohort based on the five dimensions of the EQ-5D-5L. Only those patients were included for whom data was available both at baseline and at follow-up (N = 195).

Variable	Value	Baseline	Follow-up	*P*
		%	%	
Mobility	No problems	73.3	48.7	< 0.001
	Slight problems	14.9	16.4	
	Moderate problems	7.2	20.0	
	Severe problems	3.6	13.3	
	Unable to walk around	1.0	1.5	
Self-care	No problems	85.6	82.1	0.366
	Slight problems	8.7	5.6	
	Moderate problems	2.6	6.2	
	Severe problems	2.1	5.1	
	Unable to wash or dress	1.0	1.0	
Activity	No problems	79.0	66.2	0.002
	Slight problems	11.8	8.2	
	Moderate problems	4.6	13.8	
	Severe problems	3.6	8.7	
	Unable to do usual activities	1.0	3.1	
Pain	No pain/discomfort	62.1	37.4	< 0.001
	Slight pain/discomfort	20.0	23.6	
	Moderate pain/discomfort	12.8	26.7	
	Severe pain/discomfort	4.1	11.8	
	Extreme pain/discomfort	1.0	0.5	
Anxiety	Not anxious/depressed	74.9	69.7	0.021
	Slightly anxious/depressed	15.9	11.3	
	Moderately anxious/depressed	4.6	10.8	
	Severely anxious/depressed	3.1	4.6	
	Extremely anxious/depressed	1.5	3.6	

For the overall cohort, the mean EQ-5D index values were 0.86 ± 0.26 at baseline and 0.79 ± 0.27 at follow-up. The mean EQ VAS of the overall cohort was 68.5 ± 20.2 at baseline and 68.6 ± 21.8 at follow up (Table 1).

Participants in the lowest educational level category reported a worse HRQoL at baseline and follow-up according to the EQ-5D index values (Table 5). According to the EQ VAS, patients with lower educational attainment had a worse health state at follow-up. Importantly, while the health state of participants with a lower education level worsened one year post-AMI compared to the baseline, the health state of patients with a higher education level actually improved, hereby showing an opposite trend.

**Table 5:** Health-related quality of life and self-perceived health status by socioeconomic factors, baseline and follow-up.

Variable	Group 1	Group 2	Group 3	*P* value
A. Education level				
EQ-5D index value BL	0.805 ± 0.310	0.907 ± 0.182	0.879 ± 0.274	0.005
EQ-5D index value FU	0.730 ± 0.276	0.801 ± 0.287	0.855 ± 0.229	0.01
Change EQ-5D index value BL-FU	−0.096 ± 0.317	−0.125 ± 0.263	−0.070 ± 0.213	0.17
EQ VAS BL	66.2 ± 21.1	70.5 ± 20.0	70.7 ± 16.9	0.35
EQ VAS FU	61.9 ± 21.3	70.5 ± 21.3	75.5 ± 19.9	0.002
Change EQ VAS BL-FU	−7.7 ± 26.7	−2.8 ± 22.9	4.0 ± 23.1	0.048
B. Occupational qualification				
EQ-5D index value BL	0.745 ± 0.381	0.876 ± 0.231	0.899 ± 0.233	0.37
EQ-5D index value FU	0.613 ± 0.350	0.811 ± 0.261	0.823 ± 0.236	0.03
Change EQ-5D index value BL-FU	−0.124 ± 0.448	−0.097 ± 0.242	−0.114 ± 0.234	0.92
EQ VAS BL	65.6 ± 18.9	68.4 ± 20.9	73.4 ± 15.2	0.25
EQ VAS FU	52.2 ± 24.7	70.8 ± 19.9	71.3 ± 21.2	0.004
Change EQ VAS BL-FU	−13.2 ± 30.7	−0.9 ± 23.2	−3.3 ± 24.0	0.11
C. Financial situation				
EQ-5D index value BL	0.858 ± 0.268	0.880 ± 0.232	0.921 ± 0.153	0.71
EQ-5D index value FU	0.717 ± 0.363	0.794 ± 0.263	0.810 ± 0.258	0.64
Change EQ-5D index value BL-FU	−0.140 ± 0.436	−0.085 ± 0.250	−0.111 ± 0.229	0.94
EQ VAS BL	71.8 ± 20.6	71.5 ± 19.4	70.6 ± 19.9	0.98
EQ VAS FU	62.1 ± 22.1	67.2 ± 21.5	73.2 ± 21.6	0.05
Change EQ VAS BL-FU	−11.0 ± 25.7	−5.0 ± 23.7	2.6 ± 23.5	0.02

Mean ± standard deviation. Group 1: No more than seven years of school attendance, elementary or secondary school, no school-leaving certificate. Group 2: Higher secondary school. Group 3: A-levels. BL, baseline. FU, Follow up.

Mean ± standard deviation. Group 1: No occupational qualification. Group 2: Vocational education and training. Group 3: College education. BL, baseline. FU, Follow up.

Mean ± standard deviation. Group 1: Financial situation is insufficient. Group 2: Satisfactory or adequate financial situation. Group 3: Good or very good financial situation. BL, baseline. FU, Follow up.

Participants with a lower occupational qualification reported a worse HRQoL and health state at follow-up, which was not statistically significant at baseline (Table 5).

For the financial situation, similarly to the educational level, the health state of participants with a poor financial situation worsened one year post-AMI compared to the baseline while the health state of patients with a good or very good financial situation improved (Table 5).

## Discussion

This study investigated differences in HRQoL in patients admitted for an AMI before admission and one year post-AMI depending on socioeconomic status (SES), estimated using educational level, occupational qualification, and financial hardship as indicators for SES.

As our main findings, we demonstrate that patients with lower educational attainment had a worse HRQoL estimated from the EQ-5D index values if compared with patients with a higher education level both before admission and one year post-AMI. Importantly, while the health state of participants with a lower education level worsened one year post-AMI compared to the baseline, the health state of patients with a higher education level actually improved, by that showing an opposite trend. This was the same for financial hardship. Concerning clinical presentation, patients with lower educational attainment were more likely to report dyspnea at hospital admission, had a worse renal function, and were more likely to have a history of hypertension. For the overall cohort, the health state worsened in terms of the mobility, activity, pain, and anxiety/depression domains but not the self-care domain one year post-AMI.

In general, the SES of a patient has a significant and measurable impact on cardiovascular health-related outcomes, as individuals of low SES suffer from a substantial burden of cardiovascular disease (CVD), including increased event rates and poorer outcomes^19^. Several factors can be used as surrogate markers to estimate the SES of an individual person. Out of these, four markers for the SES are associated with CVD in high-income countries: income (or the financial situation), educational attainment, employment status, and environmental factors^19^. Among these, education was previously found to be the strongest and most consistent socioeconomic indicator of cardiovascular outcomes^20,21^. The education level of individual patients is rarely changing over the time and has a determining effect on future social status and health^22^. Education seems to have a mediating nature with numerous profound interrelations with health status and disease recovery and we have previously reported about the limited evidence on the educational level and its impact on health outcomes other than mortality following AMI^23^.

Several studies identified educational attainment as a powerful predictor of cardiovascular morbidity and mortality: in patients hospitalized with an AMI, lower educational attainment was associated with a higher risk of adverse events following discharge^24–27^. Furthermore, a low educational level is associated with an increased risk of reinfarction^28^ and heart failure^12^. In line with these findings, a higher level of education correlates with lower levels of depression^29^. From the data of the German Federal Health Survey (*Bundesgesundheitssurvey*, DEGS) of the years 2008–2011, it was found that the prevalences of smoking, obesity, diabetes mellitus, depressive symptoms, diagnosed depressions, and physical inactivity are significantly higher in patients with lower SES^30–32^.

We have previously reported a significantly lower AMI hospitalization rate in areas with intermediate SSD compared to the highly deprived areas within Brandenburg^5^, reflecting the link between social determinants of health and CVD risk. In line with these findings, we were able to demonstrate a higher rate of diabetes mellitus within our study population if compared to the average for German patients who received a PCI in the year 2016^33^. However, the prevalences of arterial hypertension and diabetes mellitus within our study population are consistent with the data on the prevalence of cardiovascular risk factors in existing coronary heart disease from primary care^34^.

Similar health gradients can be observed in different diseases: Tetzlaff et al. found a widening socioeconomic gap in cancer mortality in Germany, associated with larger declines in cancer mortality in areas with less SSD. At the same time, mortality rates for certain cancers have increased over the recent years in areas with high SSD^35^. We have previously demonstrated a comparable observation for CVD, with an increased exposure to chronic oxidative stress and inflammation in regions with high and very high SSD, which depicts an important mechanism in the pathogenesis of CVD^36^. Moreover, chronic inflammation, immune and renal dysregulation appear to be important mediators of the effect of a lower educational level on the risk of cardiovascular and all-cause mortality in patients with established coronary artery disease in the United States^37^, which may also play an important role in the development of other diseases and in various cohorts, and may be an important biological mechanism of adverse social determinants of health.

Lately, self-perceived health factors including the HRQoL following an acute coronary event have been increasingly studied to assess outcome beyond classical endpoints such as major adverse cardiac events (MACE) including AMI, stroke, cardiovascular death, hospitalization for unstable angina or revascularization procedures, and heart failure. It is widely recognized that socioeconomic and demographic factors have a significant influence on the quality of life within the general population and among various cohorts^38,39^, however, long-term data of patients suffering from CVD is lacking. Furthermore, only few studies considered socioeconomic factors as part of their analyses, although this approach seems to be particular important as quality of life impacts prognosis: Pocock et al. assessed associations of HRQoL and patient characteristics, healthcare utilization, adverse cardiovascular events, and mortality in post-AMI patients, showing that a poor HRQoL was associated with higher risks of all-cause mortality and MACE^7^. It should therefore be recognized that HRQoL is linked to prognosis in patients with AMI and that these findings have important implications for cardiac rehabilitation care for post-AMI patients in order to enhance the HRQoL.

In 2017, Tchicaya and Lorentz measured HRQoL among patients with coronary artery disease (CAD) five years after a coronary angiography. As a main finding, a lower education level and impaired living conditions negatively affected the HRQoL^40^.

The present study shows clear inequalities in HRQoL based on SES indicators, which increased within one year after an AMI. To achieve further success in the treatment of patients after an AMI, patient-centered care that takes socioeconomic factors into account is essential. For patients with an acute coronary syndrome (ACS), it has been shown that improved individualized care positively impacts medication adherence^41^. Another study has shown that a specific patient-centered treatment plan can improve the health of patients after an ACS, especially in patients with a lower education level^42^. Research that considers local medical infrastructure and the importance of SSD is needed.

Beyond CVD, in a large global cohort of cancer survivors, several socioeconomic factors were associated with quality of life, with lower educational attainment and lower income being negatively correlated with physical and mental HRQoL^43^. Therefore, determinants of the HRQoL should be identified when evaluating strategies for secondary prevention.

Limitations of our study include a 29.9% loss of follow-up. This includes all patients who we were unable to reach by telephone on multiple occasions (on at least three different days). Reasons may include death, change of phone number, longer vacation or inability to answer the phone due to the health condition after one year. Another limitation of our study is that HRQoL was self-reported and is not an objective measure of health. As this is a single center study in a rural-suburban area in eastern Germany in patients suffering from AMI, our findings may not be generalizable to other settings, patients, and populations. As the study was conducted during both the pre-COVID-19 era as well as the time of the first and second infection wave, the COVID-19 pandemic may have played a role in HRQoL assessment, especially for the depression/anxiety EQ-5D dimension. A further limitation of this study is that only a subset of AMI patients who presented at our hospital—specifically, those who provided written informed consent and were able to complete the study at both time points—were included. These individuals were likely to be generally healthier and less clinically impaired by AMI. As the follow-up of the patients consisted of a short telephone interview focusing on HRQoL assessment, clinical data and detailed information on the conditions of the patients at one year were not part of the present study. In order to carry out a comparative analysis of the baseline and follow-up HRQoL, we also did not adjust the baseline HRQoL for potential confounders, as otherwise it would not have been possible to compare the values. We acknowledge this to be a limitation to our results.

## Conclusion

To summarize, we provide first data that lower educational attainment and lower occupational qualification negatively affect the HRQoL already within the first year in patients undergoing emergent coronary angiography due to an AMI. Taken together, there is strong evidence that a lower SES is associated with morbidity and mortality in patients with CAD, but is also associated with the HRQoL. A worsening health state over the course of one year in patients presenting with an AMI is particularly evident in those with low educational attainment but also in those experiencing a financial situation that is insufficient, hereby showing an opposing trend when compared to patients with a higher educational level and patients with a good or very good financial situation in which the health state improves over the course of one year.

## Data Availability

Data can be made available for researchers who meet the criteria for access to confidential data upon reasonable request to the corresponding author.
